# A *de novo* germline mutation in *MYH7* causes a progressive dominant myopathy in pigs

**DOI:** 10.1186/1471-2156-13-99

**Published:** 2012-11-15

**Authors:** Leonardo Murgiano, Imke Tammen, Barbara Harlizius, Cord Drögemüller

**Affiliations:** 1 Institute of Genetics, Vetsuisse Faculty, University of Bern, Bremgartenstrasse 109a, Bern, 3001, Switzerland; 2 ReproGen, Faculty of Veterinary Science, The University of Sydney, Camden, NSW, 2570, Australia; 3 Institute for Pig Genetics BV, PO Box 43, Beuningen, 6640 AA, The Netherlands

## Abstract

**Background:**

About 9% of the offspring of a clinically healthy Piétrain boar named ‘Campus’ showed a progressive postural tremor called Campus syndrome (CPS). Extensive backcross experiments suggested a dominant mode of inheritance, and the founder boar was believed to be a gonadal mosaic. A genome-scan mapped the disease-causing mutation to an 8 cM region of porcine chromosome 7 containing the *MHY7* gene. Human distal myopathy type 1 (MPD1), a disease partially resembling CPS in pigs, has been associated with mutations in the *MYH7* gene.

**Results:**

The porcine *MYH7* gene structure was predicted based on porcine reference genome sequence, porcine mRNA, and in comparison to the human ortholog. The gene structure was highly conserved with the exception of the first exon. Mutation analysis of a contiguous genomic interval of more than 22 kb spanning the complete *MYH7* gene revealed an in-frame insertion within exon 30 of *MYH7* (c.4320_4321insCCCGCC) which was perfectly associated with the disease phenotype and confirmed the dominant inheritance. The mutation is predicted to insert two amino acids (p.Ala1440_Ala1441insProAla) in a very highly conserved region of the myosin tail. The boar ‘Campus’ was shown to be a germline and somatic mosaic as assessed by the presence of the mutant allele in seven different organs.

**Conclusion:**

This study illustrates the usefulness of recently established genomic resources in pigs. We have identified a spontaneous mutation in *MYH7* as the causative mutation for CPS. This paper describes the first case of a disorder caused by a naturally occurring mutation in the *MYH7* gene of a non-human mammalian species. Our study confirms the previous classification as a primary myopathy and provides a defined large animal model for human MPD1. We provide evidence that the CPS mutation occurred during the early development of the boar ‘Campus’. Therefore, this study provides an example of germline mosaicism with an asymptomatic founder.

## Background

In 1988, a commercial center for artificial insemination in southern Germany recognised a number of piglets affected by a progressive tremor. Those piglets were identified as being the progeny of a clinically normal Piétrain boar named ‘Campus’ used for artificial insemination. Affected pigs started showing clinical signs of the disorder at 2–9 weeks of age: muscular tremors starting in the hind limbs followed by tremors in the fore limbs briefly after. Tremors were present while walking (with an impaired gait) or standing only and absent at rest (Additional file 1). The clinical course of the so called ‘Campus’-syndrome (CPS) was progressive and the tremors were more severe in older animals, which became quickly exhausted [1,2]. The pigs were highly stress-susceptible and most of them died from heart attack before maturity. Muscle fiber degeneration and regeneration as well as interstitial fibrosis were observed, and this was associated with primary myopathy [3,4]. Intriguingly, macroscopic and light microscopic examinations of brain and peripheral nerves detected no morphological alteration [2]. In addition, neurophysiological studies with quantitative electromyography did not determine any myopathic changes and motor nerve conduction velocity appeared to be unaltered [5].

For further characterization of the disorder, the boar ‘Campus’ was mated with clinically healthy sows of six different pig breeds [1]. The offspring of matings between ‘Campus’ and 7 healthy sows showed about 9.3% (26 out of 270) affected piglets. Both sexes were affected equally. However, 10 out of 15 of the offspring of three affected sows showed clinical signs of the syndrome. This strongly indicated an autosomal dominant mode of inheritance, where the founder ‘Campus’ was believed to be a gonadal mosaic [1,5]. Using animals from the backcross experiment, a genome-scan with 254 porcine microsatellite markers across all porcine autosomes was performed to map the CPS locus to a region of approximately 8 cM on porcine chromosome (SSC) 7 [1]. At that time, a comparison of human and pig genome maps indicated homology to human chromosome (HSA) 15q and 14q11-q13 which contains the human dominant distal myopathy type 1 (MPD1) locus [1,6]. Clinical signs of MPD1 partially resemble CPS and subsequently, myosin VII (*MYH7*) gene mutations have been reported to be associated with MPD1 in humans [7].

The molecular analysis of the functional and positional candidate gene was hindered by the lack of sequence data for porcine *MYH7.* However, availability of the reference pig genome sequence facilitated the sequencing of the entire porcine *MYH7* gene described in this paper. We identified the CPS causing mutation which confirmed the suggested dominant mode of inheritance and the gonadal mosaicism of the founder boar.

## Results

As the annotation of the porcine genome is still in progress, we inferred the porcine *MYH7* gene structure manually in comparison to the corresponding human *MYH7* gene. Using long-range PCRs we sequenced the whole gene, exons and introns. We characterized the genomic structure of the porcine *MYH7* gene and performed mutation analysis. These analyses indicated that the porcine *MYH7* gene consists of 40 exons separated by 39 introns and spans 22 kb compared to 39 exons over 21.5 kb in human *MYH7* (NCBI build 37.3) due to an additional untranslated 5^′^-exon in pigs (Additional file 2: Figure S1). As the current porcine genome assembly (build 10.2) contained a gap including the sequence of *MYH7* intron 19, we submitted the experimentally derived genomic sequence of 573 bp containing 281 bp sequence of intron 19, and flanking exon 19 and 20 sequences into the EMBL nucleotide database (Acc. HE663068). All splice donor/splice acceptor sites conform to the GT/AG rule. The porcine transcript contains an open reading frame of 5,808 bp encoding a protein of 1,935 amino acids.

**Figure 1:**
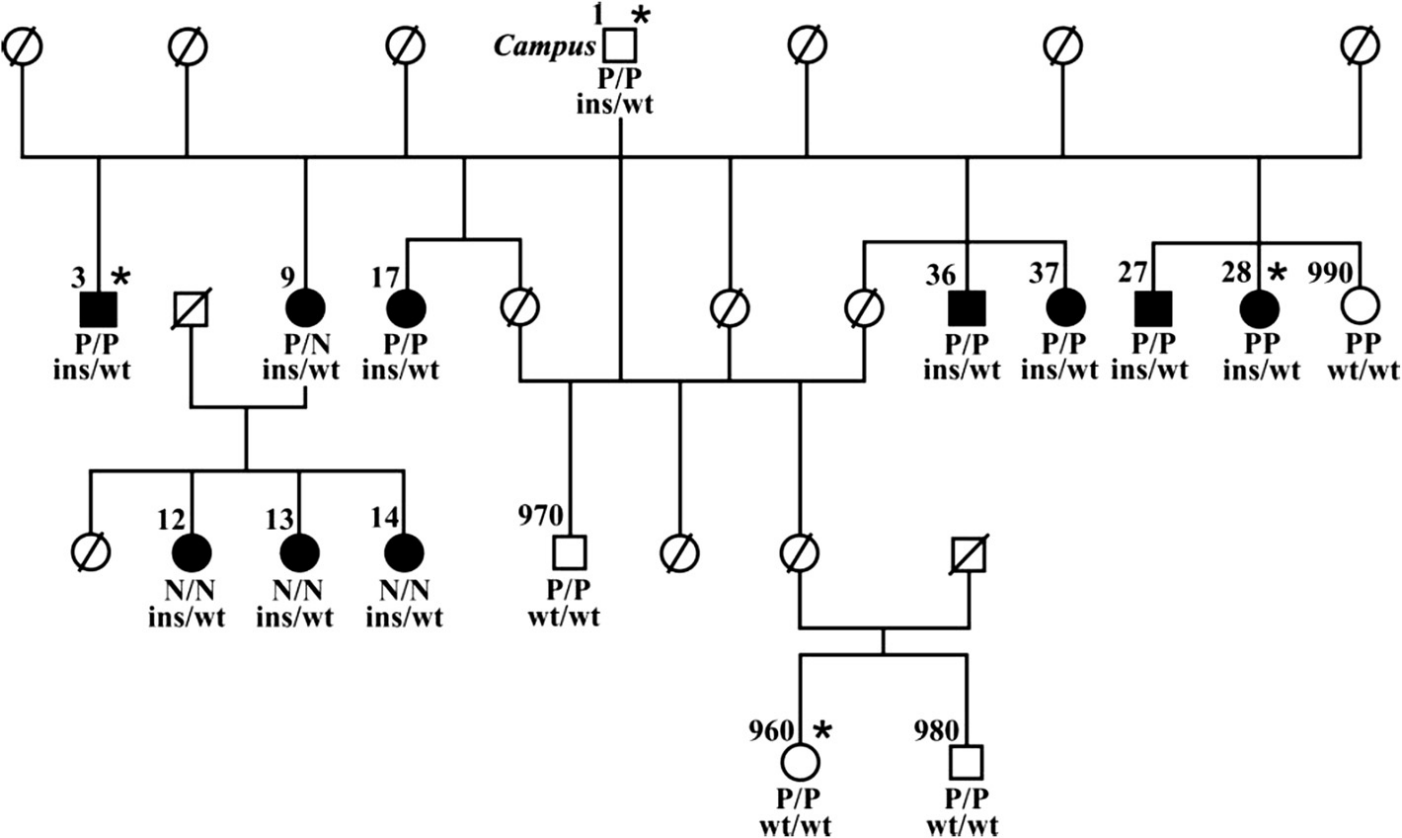
**‘Campus’ syndrome pedigree (modified from Tammen et al.**[1]**)**. DNA samples were available for this study only from 15 numbered pigs. Note that the founder boar “Campus” was not affected. Four animals used for re-sequencing and mutation screening are indicated by asterisk. The genotypes for the *RYR1* mutation causing malignant hyperthermia [8] (P = mutation, N = wildtype) and the *MYH7* c. 4320_4321insCCCGCC genotypes (ins = insertion, wt = wildtype) are given below the symbols.

**Figure 2:**
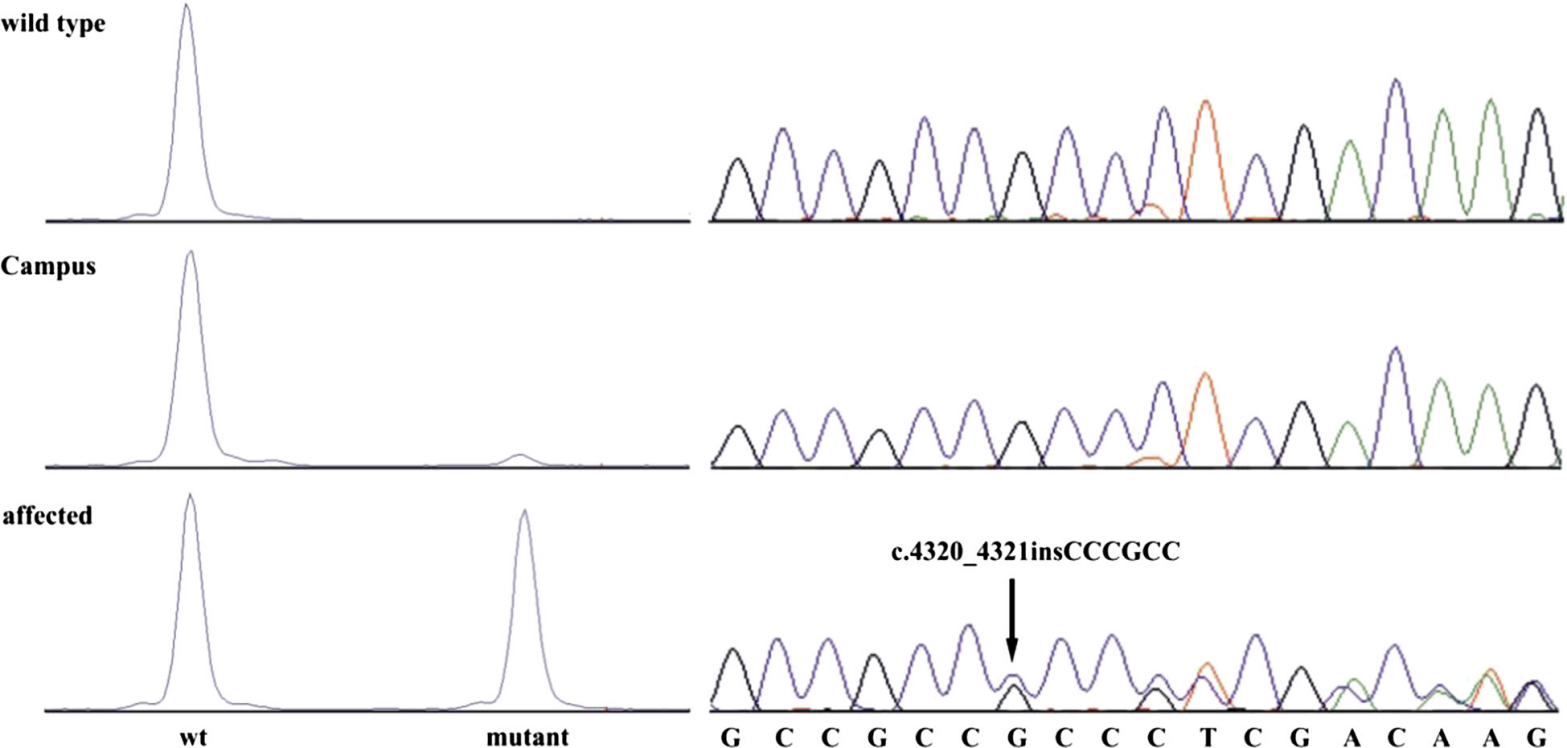
**Electropherograms of the *MYH7 *c. 4320_4321insCCCGCC mutation.** Representative sequence traces of PCR products amplified from genomic DNA of 3 pigs with the different genotypes are shown on the right hand side. The presence of the mutation can be directly visualized by fragment size analysis of a fluorescently labeled PCR product containing the mutation as shown on the left. Note that the mutant allele with the 6 bp-insertion is present in heterozygous form in ‘Campus’ and in CPS affected piglets at different ratio.

We re-sequenced a contiguous genomic interval of more than 22 kb spanning the complete *MYH7* gene including all coding exons in four pigs (Campus, two affected individuals and a healthy control, Figure 1). Nine sequence polymorphisms (Additional file 3: Table S1) were found in the examined pigs as compared to the *MYH7* reference sequence of the pig genome assembly (build 10.2). Only one of these sequence variants, the six base-pair insertion c.4320_4321insCCCGCC located in exon 30 of the *MYH7* gene (Figure 2), was perfectly associated with the CPS phenotype in the four animals (Figure 1). We genotyped all 14 available animals in the experimental pedigree for this insertion and found a perfect association of the mutant insertion-allele with the CPS phenotype. All affected animals were heterozygous for the mutant allele and none of the controls carried this allele (Figure 1). The mutant allele was absent from 88 normal pigs from 8 genetically diverse breeds including wild boars.

**Figure 3:**
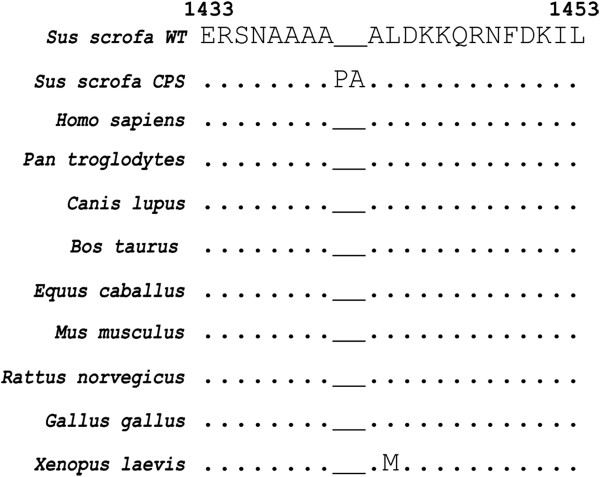
**Multiple sequence alignment of the MYH7 protein in the region of the mutation.** Note the nearly perfect conservation of the surrounding protein sequence. The observed mutation in CPS affected pigs is indicated by additional two residues. Identical residues are indicated by a point beneath the alignment, while different amino acids are reported (the methionine in *Xenopus laevis* is a very similar aminoacid).

The predicted consequence of the in-frame insertion is an insertion of two amino acids (p.Ala1440_Ala1441insProAla) in a very highly conserved protein region (Figure 3).

Initially, sequencing didn’t show the presence of the identified mutation in the assumed founder ‘Campus’ (Figure 2). Subsequently, we established a more sensitive approach using a fluorescently labeled PCR primer and fragment analysis which showed that the boar clearly carries the mutant allele at low level in comparison to the wild type allele (Figure 2). Genotyping of the *MYH7* exon 30 mutation in various organs (liver, kidney, skeletal muscle (hind and fore limb), testicle, epidydimis, brain stem, and blood) of ‘Campus’ showed the presence of both the wild type allele and a smaller sized peak for the mutant allele in all available samples (Figure 2, the relative area ratio for each organ is reported in Table 1).

**Table 1:** Percentage of mosaicism in different organs in mosaic boar ‘Campus’

**Tissue**	**Percentage of mosaicism (from area ratio)**	**Germinal layer**
Liver	17.2	Endoderm
Testicle	8.6	Mesoderm, germline (entodermal)
Blood	3.4	Mesoderm/Mesenchyme
Kidney	6.0	Mesoderm
Hindlimb muscle	5.4	Mesoderm
Forelimb muscle	11.6	Mesoderm
Epidydimis	5.2	Mesoderm
Brain stem	6.6	Neuroectoderm

A disease with a stress susceptibility phenotype, malignant hyperthermia (MHS) syndrome, is common in Piétrain pigs. This is an inherited myopathy caused by a mutation in the *RYR1* gene in which skeletal muscle contracture with hypermetabolism and elevation in body temperature are triggered by stress or experimentally by inhalational anesthetics and skeletal muscle relaxants [8]. To assess the influence of the *RYR1* locus, all animals and every ‘Campus’ organ sample analyzed in this study have been tested for the MHS causing *RYR1* mutation. Among the 10 pigs affected with CPS, six were homozygous for the MHS mutation, one was heterozygous and three were homozygous for the wild type allele (Figure 1).

## Discussion

After successful linkage mapping of the dominantly inherited CPS mutation, the molecular analysis of the positional and functional candidate gene *MYH7* was assisted by the availability of the porcine reference genome sequence. The clustering of two highly similar genes (*MYH6* and *MYH7*) within the region of interest made a specific mutation analysis difficult without proper gene annotation. Therefore, we initially performed a manually generated gene annotation and confirmed the high sequence homology between *MYH7* and the neighboring *MYH6* gene. The *MYH6* gene encodes the alpha heavy chain subunit of cardiac myosin, and mutations in *MYH6* cause congenital heart muscle defects in humans [9]. We choose the slow skeletal muscle fiber myosin heavy chain gene *MYH7* gene as candidate for CPS as it is predominantly expressed in the fetal skeletal muscle and after birth in skeletal muscle, MYH7 is expressed specifically in type 1 fibers [10]. In the heart, it is only switched on under conditions of thyroid hormone depletion / replacement and in response to physical stress [11]. Van Rooij et al. [11] showed that adult onset of expression of *MYH7* in the heart is regulated by a microRNA (miRNA 208), encoded by intron 27 of *MYH6*, at stress*.* These findings point towards MYH7 as candidate gene because several affected CPS pigs died after stress from a sudden heart attack.

Mutation analysis for CPS detected a perfect association between the 6 bp insertion in exon 30 of *MYH7* and the disease phenotype. The insertion (c.4320_4321insCCCGCC) is predicted to prolong the encoded protein by the insertion of the two amino acids alanine and proline (p.Ala1440_Ala1441insProAla). The insertion occurs 60 amino acids before the myomesin binding domain region of the filament backbone, in the myosin tail in the C-terminal end of the protein, which has the purpose of supporting the head in the contraction movement and creates a coiled-coil structure with another paired myosin molecule (Additional file 2: Figure S1). The insertion occurs in the myosin tail of the MYH7 protein characterized by a stretch of alanines (NAAAA**PA**ALDKK, the insertion is bolded and underlined). This protein region is highly conserved across species (Figure 3). The insertion of proline in a sequence forming a coiled coil very likely explains the observed disease phenotype. These structures have an important role as main structural feature known to dimerize myosins to form the molecular motors of the musclular fiber [12] It has been pointed out that proline is generally destructive for the formation of a coiled coil structure [13] both in the *a* and *d* position. Using Marcoil, a software that predicts existence and location of potential coiled-coil domains in protein sequences [14,15], we observed that in comparison to the wild type, the insertion we observed caused a drop to zero of the probability of formation of the coiled coil, suggesting a disruptive effect of the mutation. Furthermore, additional effects of the mutation on the overall structure of the coil (after the heptad is shifted by two amino acids) cannot be excluded. In a helical coiled-coil, residues in *a* and *d* position are typically hydrophobic and create a seam along the interface of the coiled-coil, whereas residues in the *e* and *g* positions interact electrostatically to stabilize the coiled-coil. Residues in the outer positions such as *b*, *c*, and *f* are typically charged and mediate the interactions between coiled-coils. Blair et al. (2002) pointed out that for some of the disease causing mutations the pathomechanism might be due to disruption of interactions with other sarcomeric proteins and complexes and not just due to changes to the coiled coil structure itself [16]. The myosin head alone is sufficient to generate force in the fiber [17]. Mutations in the coiled coil could cause a defect in the transmission of force to the thick filament array. They could either affect assembly of the thick filament or stability of the protein [18]. See Additional file 4: Figure S2, Additional file 5: FigureS3, and Additional file 6: Table S2 for further details.

In both human MPD1 and porcine CPS, the disease allele is dominant. When comparing the porcine *MYH7* mutation causing CPS to known Laing early onset distal myopathy (MPD1) causing mutations in humans, a proper assessment of the structure-function relationship of *MYH7* mutations remains challenging: there is no clear association between the position of the mutation and resulting pathological effects. Mutations at different positions in the *MYH7* gene can lead to the same disease, however, mutations in the same position have been associated with different diseases [19-23]. Mutations affecting the coiled coil are reported to cause HMC, dilated cardiomyopathy (DCM), LVNC, and MPD1 [17] (Figure 1B).

The position of the mutation compared to human reports could seem slightly “off-target” compared to human reports: The range of the MPD1 associated mutations reported are located between amino acid 1500 and 1729 of MYH7 encoded by *MYH7* exons 32 to 36 [20]. Nevertheless, cases of MPD1 caused by mutations out of this range are reported, as an example in *MYH7* exon 40 [24], or a case of distal myopathy caused by a *MYH7* exon 16 mutation in the head of myosin showing associated cardiomyopathy [25].

Another factor worthy of consideration is the chemical properties of the amino acids inserted. Meredith et al. (2004) compared the nature of mutations in HCM and MPD1, and pointed out that mutations associated with HCM had no significant effect on the probability of coiled coil formation, whereas for MPD1 it was evident in all the mutations studied had some disruptive effect on the coiled coil formation [7]. These results suggest that the observed clinical heterogeneity of human diseases caused by *MYH7* mutations in the myosin rod could be related to different effects on stability of the mutant MYH7 residue [26]. For example, two independent *MYH7* exon 32 mutations affecting the same amino acid (R1500P and R1500W) cause different diseases [26]. The proline substitution causes MPD1 and seems to not alter the coiled coil structure, but to heavily destabilize it with greater decrease in filament stability. On the other hand, the tryptophan substitution causes HCM and shows a greater decrease in thermodynamic stability. In the same paper, the authors provide a possible explanation for the etiology of different clinical signs and diseases, suggesting that different mutations can lead to different destiny of the altered fiber in cardiac or skeletal muscle and hence explain the possibility of lack of HCM symptoms in MPD1 patients and vice versa [11,26].

The CPS causing mutation in exon 30 affects the myosin tail domain of porcine MYH7. It can be assumed that the insertion causes a disruption of the myosin function, and this would suggest that CPS is similar to human MPD1 in regards of the disease pathomechanism. Furthermore, the early onset, the weakness in the hip and shoulder muscles in the affected pigs (Additional file 1), as well the lack of increase in creatine kinase are similar to most forms of human MPD1 [2,21]. In addition, the progressive tremor in CPS affected pigs resembles the reported progressive weakness in MPD1 patients [27]. The low life expectancy of pigs affected by CPS could be related to human HCM, which is a cause of sudden death in otherwise normal individuals. Several mutations located in exon 30 of human *MYH7* lead to HCM [16]. However, hearts of pigs affected by CPS showed no cardiac hypertrophy [2]. A possible background effect of the *RYR1* genotype on stress susceptibility in CPS affected pigs has been ruled out. There is not association between *RYR1* mutation and the phenotype, as well with the piglet low life expectancy (Figure 1). Therefore, we suggest that the detected *MYH7* mutation fully explains the disease phenotype in the CPS affected piglets.

We suggest that ‘Campus’ is confirmed as a founder mosaic, as it was shown for ‘Solid Gold’ for ovine callipyge [28] In fact, as in the case of ‘Solid Gold’, genotyping of 21 autosomal microsatellites markers pointed out no presence of three or four alleles for ‘Campus’, so we excluded microchimerism. On the other hand, in regard of the specific mutation, the genetic analysis of different organs from ‘Campus’ showed that the mutant *MYH7* allele is present in all analyzed samples at variable levels ranging from 3.4 to 17.2% (Table 1). These results revealed that ‘Campus’ is a gonadal and somatic mosaic. With regard to the observed variability between organs, it is relevant to notice that the examined organs contain a full set of different tissues. As an example brain neurons and glial cells develop from the neuroectoderm, but the brain as such also contains blood vessels of mesenchymal origin and minute amounts of connective tissue - along the vessels. Similarly, hepatocytes are derived from the entoderm but the liver also contains connective tissue and blood vessels. Thus in the assays, one would always end up with a mixture of tissues from different embryonic origin. Hence is it possible that different sampling could lead to different results because of a granularity of the mosaicism recognizable with other methods. Germline mosaicism is a relatively frequent mechanism in the origin of genetic disorders [29]. Depending on various factors, such as the gene involved and/or the degree of mosaicism, the carrier of somatic and germline mosaicism may be asymptomatic or may present with various symptoms of the disease [29]. There are two possibilities for the existence of such a mosaicism: one is that the mutation occurs in a germ cell that continues to divide. The other possibility is that the mutation occurs very early in a somatic cell before the separation to germinal cells and is therefore present both in somatic and germinal cells [29]. This seems to be the most likely explanation for the situation of ‘Campus’ as he carries the mutant allele in somatic and germinal cells, although he didn’t show clinical signs like the affected offspring.

## Conclusions

This study elucidates the molecular basis of a spontaneous lethal mutation initially detected by commercial pig breeders. Unraveling the mutation was facilitated by the largely improved genomic resources in pigs. This paper describes the first case of a skeletal muscular disorder caused by a naturally occurring mutation in the *MYH7* gene of a non-human mammalian species. The genetically characterized porcine CPS phenotype may represent an interesting large animal model for human MPD1. The dominantly inherited CPS disorder belongs to the group of cases where mosaicism was suspected in one of the asymptomatic parents. We provide evidence that the mutation event can be assigned to an early stage before the differentiation of germinal cells during embryonic development.

## Methods

### Animals

We collected liver tissue samples from 15 members of the experimental pedigree used for the initial linkage mapping [1] including the founder boar ‘Campus’, ten piglets affected with CPS, and four healthy offspring (Figure 1). In addition, for ‘Campus’ we collected tissue samples of kidney, skeletal muscle (hind, and fore limb), testicle, epidydimis, brain stem, and EDTA stabilized blood. Furthermore, we sampled genomic DNA of 88 unrelated male control pigs from eight different breeds (Duroc (n = 1), Husumer (n = 1), Landrace (n = 1), Large White (n = 1), Meishan (n = 1), Pietrain (n = 9), Swiss Landrace (n =66), Wild boar (n = 9)) for the genotyping of the *MYH7* exon 30 insertion. We genotyped the *RYR1* mutation associated with malignant hyperthermia in pigs as described previously [8].

All animal work has been conducted according to the national and international guidelines for animal welfare. There is no permit number as this study is not based on an invasive animal experiment. Pigs were slaughtered at commercial abattoirs for sample collection; there was no “animal experiment” according to the legal definitions in Germany.

### Sequence analysis

The human reference *MYH7* mRNA [GenBank: NM_000257] was used as query in cross-species BLAST searches against pig genome assembly (build 10.2), porcine mRNA, and EST sequence databases respectively. A single porcine genomic SSC 7 contig of 319,923 bp was identified [GenBank: NW_003610693]. Furthermore, a porcine reference mRNA sequence of 5,996 bp [GenBank: NM_213855] and a 5^′^UTR containing porcine EST [GenBank: DN132389] were available. The exact porcine genomic structure was determined using Spidey [30] and Otterlace [31].

### Mutation analysis

Genomic DNA was isolated using the DNeasy Blood & Tissue Kit (Qiagen) according to the manufacturer’s protocol. Prior to primer design using Primer3 [32] repetitive sequences were masked employing Repeatmasker [33]. For the mutation analysis PCR products were amplified of the founder boar, two affected piglets and a single normal piglet. PCR products were amplified using SequalPrep Long PCR Kit (Invitrogen). PCR products were sequenced on both strands using internal sequencing primers on an ABI 3730 capillary sequencer (Life Technologies) after treatment with exonuclease I and shrimp alkaline phosphatase. Sequence data were analyzed with Sequencher 4.9 (GeneCodes). Fragment size analyses for the genotyping of the *MYH7* exon 30 insertion were also performed on an ABI 3730 capillary sequencer and analyzed with the GeneMapper 4.0 software (Applied Biosystems).

## Authors' contributions

LM did the experimental work and drafted the manuscript. IT and BH provided samples, video and performed manuscript editing. CD supervised the work, analyzed the sequence data, and performed manuscript editing. All authors read and approved the final manuscript.

## Supplementary Material

Additional file 1 Video showing signs of the condition. Click here for file

Additional file 2 **Figure S1.(A)** Gene structure of human and porcine *MHY7* gene*.* The position of the identified CPS mutation is indicated by a red arrow. **(B)** Human myosin VII protein and location of known disease causing mutations within the regions encoded by exons 30 to 32. Modified from Klaassen et al. [19] (Hypertrophic cardiomyopathy (HCM), Left ventricular noncompaction (LVNC), dilated cardiomyopathy (DCM), myosin storage myopathy (MSM), Laing early onset distal myopathy (MPD1)). Click here for file

Additional file 3 **Table S1.** Polymorphisms and genotypes of 4 pigs in the region of the MYH7 gene. Click here for file

Additional file 4 **Figure S2.** Graphical representation of the coiled coil prediction on the impact of the mutation. **(A)** wildtype protein **(B)** mutant protein. A dramatic drop in probability of the formation of a coiled coil structure is expected in the latter. Click here for file

Additional file 5 **Figure S3.** Position of amino acid in the region of the MYH7 mutation. **(A)** Scheme of a generic heptad in a dimeric parallel coiled coil. Positions in the coil are strictly dictated by the chemical properties of the residues. Wide, continue red line stands for hydrophobic interaction, and dotted line for ionic ones. **(B)** Same structure in side view **(C)** Side view of amino acids in the 1431 – p.1454 positions in wildtype as predicted by MARCOIL **(D)** roles predicted by MARCOIL for mutant protein (see Additional file 6: Table S2). Is it expected that the specific pattern regarding the formation of bonds is disrupted in the specific zone. See [34]. Click here for file

Additional file 6 **Table S2.** Probability of formation of coiled coil structure and position assigned to each residue performed by MARCOIL for the wildtype and mutant MYH7. (PDF 98 kb) Click here for file
