# Supplementary material for: A de novo germline mutation in MYH7 causes a progressive dominant myopathy in pigs
Source: BMC Genet. 2012 Nov 15;13:99. doi: 10.1186/1471-2156-13-99 (PMC3542579; doi:10.1186/1471-2156-13-99)

Suppl. Figure 1 (A) Gene structure of human and porcine *MHY7* gene.

The position of the identified CPS mutation is indicated by a red arrow. (B) Human myosin VII protein and location of known disease causing mutations within the regions encoded by exons 30 to 32. Modified from Klaassen et al. [19] (Hypertrophic cardiomyopathy (HCM), Left ventricular noncompaction (LVNC), dilated cardiomyopathy (DCM), myosin storage myopathy (MSM), Laing early onset distal myopathy (MPD1))

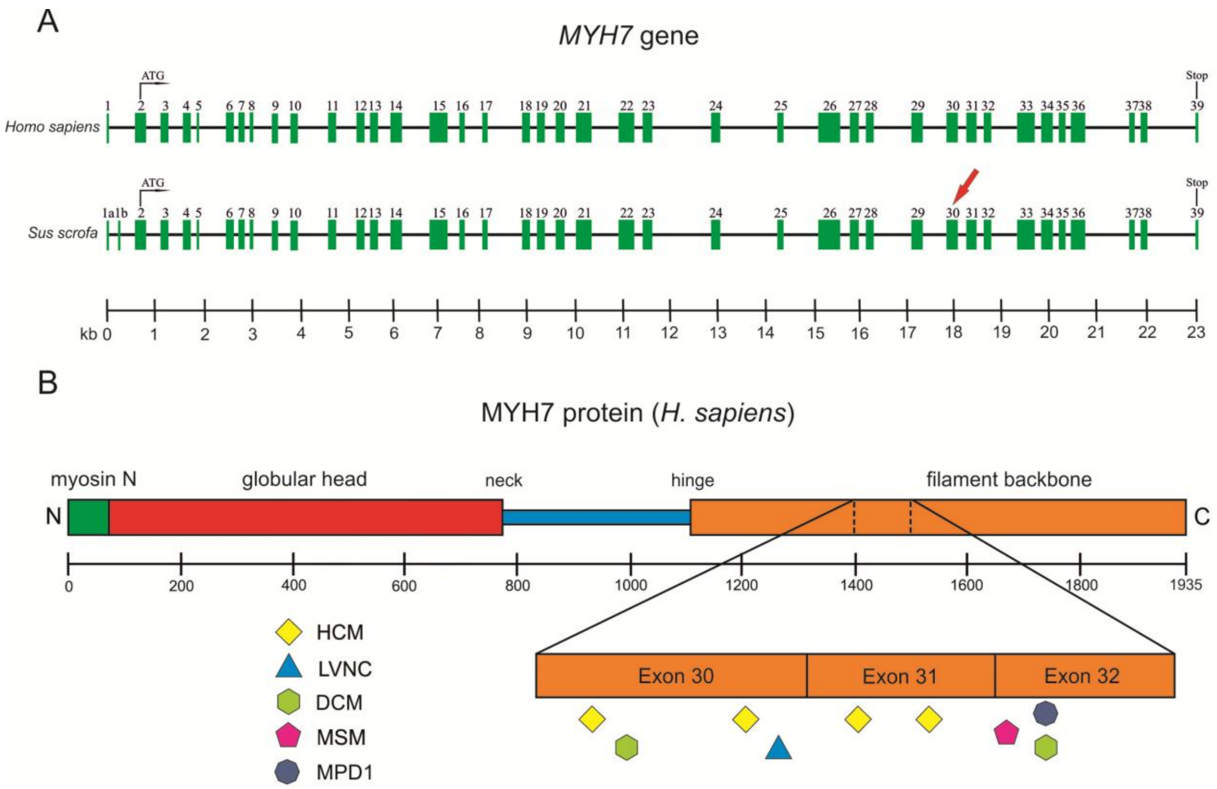

Supplement: Additional file 2 — Figure S1.(A) Gene structure of human and porcine MHY7 gene. The position of the identified CPS mutation is indicated by a red arrow. (B) Human myosin VII protein and location of known disease causing mutations within the regions encoded by exons 30 to 32. Modified from Klaassen et al. [19] (Hypertrophic cardiomyopathy (HCM), Left ventricular noncompaction (LVNC), dilated cardiomyopathy (DCM), myosin storage myopathy (MSM), Laing early onset distal myopathy (MPD1)). [file 1471-2156-13-99-S2.pdf]
