# Supplementary material for: A de novo germline mutation in MYH7 causes a progressive dominant myopathy in pigs
Source: BMC Genet. 2012 Nov 15;13:99. doi: 10.1186/1471-2156-13-99 (PMC3542579; doi:10.1186/1471-2156-13-99)

Suppl. Figure 2 Graphical representation of the coiled coil prediction on the impact of the mutation. **(A)** wildtype protein **(B)** mutant protein. A dramatic drop in probability of the formation of a coiled coil structure is expected in the latter.

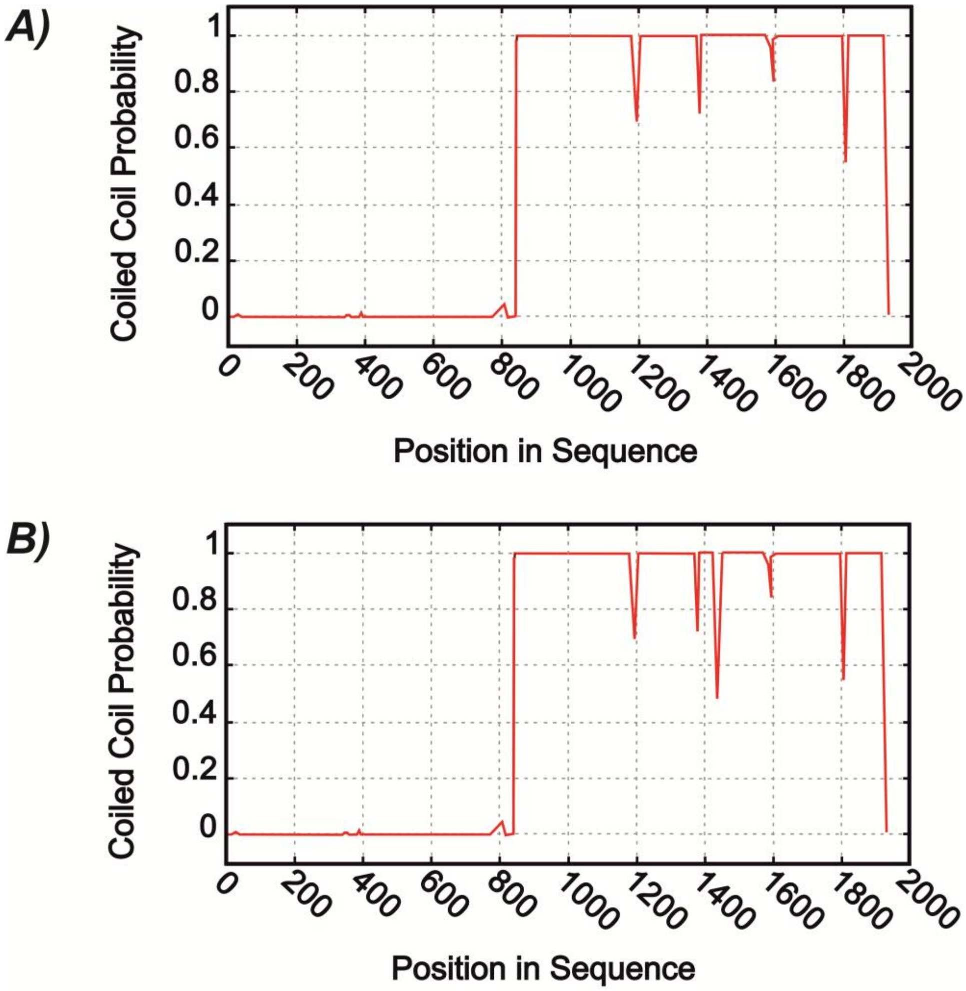

Supplement: Additional file 4 — Figure S2. Graphical representation of the coiled coil prediction on the impact of the mutation. (A) wildtype protein (B) mutant protein. A dramatic drop in probability of the formation of a coiled coil structure is expected in the latter. [file 1471-2156-13-99-S4.pdf]
