# Supplementary material for: A de novo germline mutation in MYH7 causes a progressive dominant myopathy in pigs
Source: BMC Genet. 2012 Nov 15;13:99. doi: 10.1186/1471-2156-13-99 (PMC3542579; doi:10.1186/1471-2156-13-99)

Suppl. Figure 3 Position of amino acid in the region of the MYH7 mutation.

(A) Scheme of a generic heptad in a dimeric parallel coiled coil. Positions in the coil are strictly dictated by the chemical properties of the residues. Wide, continue red line stands for hydrophobic interaction, and dotted line for ionic ones. **(B)** Same structure in side view **(C)** Side view of amino acids in the 1431 – p.1454 positions in wildtype as predicted by MARCOIL **(D)** roles predicted by MARCOIL for mutant protein (see **Suppl. Table 2**). Is it expected that the specific pattern regarding the formation of bonds is disrupted in the specific zone. See [34].

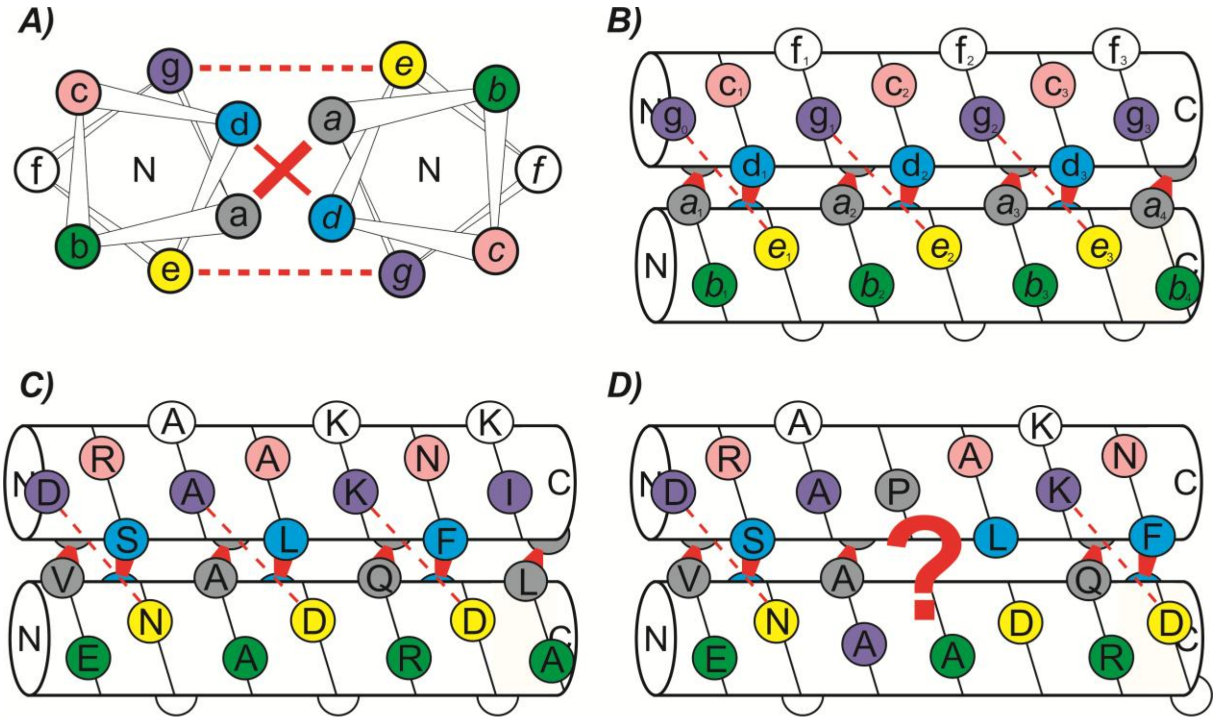

Supplement: Additional file 5 — Figure S3. Position of amino acid in the region of the MYH7 mutation. (A) Scheme of a generic heptad in a dimeric parallel coiled coil. Positions in the coil are strictly dictated by the chemical properties of the residues. Wide, continue red line stands for hydrophobic interaction, and dotted line for ionic ones. (B) Same structure in side view (C) Side view of amino acids in the 1431 – p.1454 positions in wildtype as predicted by MARCOIL (D) roles predicted by MARCOIL for mutant protein (see Additional file 6: Table S2). Is it expected that the specific pattern regarding the formation of bonds is disrupted in the specific zone. See [34]. [file 1471-2156-13-99-S5.pdf]
