# Supplementary material for: A de novo germline mutation in MYH7 causes a progressive dominant myopathy in pigs
Source: BMC Genet. 2012 Nov 15;13:99. doi: 10.1186/1471-2156-13-99 (PMC3542579; doi:10.1186/1471-2156-13-99)
Supplement: Additional file 6 — Table S2. Probability of formation of coiled coil structure and position assigned to each residue performed by MARCOIL for the wildtype and mutant MYH7. (PDF 98 kb) [file 1471-2156-13-99-S6.pdf]

Suppl. Table 2 Probability of formation of coiled coil structure and position assigned to each residue performed by MARCOIL for the wildtype and mutant MYH7.

	Wildtype			Mutant		
**Residue Position**	**Residue**	**Probability (%)**	**Coil Position**	**Residue**	**Probability (%)**	**Coil Position**
1432	V	100	a	V	89.7	a
1433	E	100	b	E	88.3	b
1434	R	100	c	R	81.4	c
1435	S	100	d	S	72.4	d
1436	N	100	e	N	69.9	e
1437	A	100	f	A	66.4	f
1438	A	100	g	A	61.4	g
1439	A	100	a	A	58.2	a
1440	A	100	b	A	53.8	g
				**P**	**48**	**a**
				**A**	**61.3**	**b**
1441	A	100	c	A	70.8	c
1442	L	100	d	L	80.5	d
1443	D	100	e	D	82.9	e
1444	K	100	f	K	86.1	f
1445	K	100	g	K	87.7	g
1446	Q	100	a	Q	88.2	a
1447	R	100	b	R	89	b
1448	N	100	c	N	90	c
1449	F	100	d	F	90.8	d
1450	D	100	e	D	92.2	e
